# 

**Published:** 1902-05

**Authors:** 


### BOOKS RECEIVED.

Saunders's Medical Hand-Atlases. Atlas and Epitomé of Otology. By Gustav Brühl, M. D., of Berlin, with the collaboration of Professor Dr. A. Politzer, of Vienna. Edited, with additions, by S. MacCuen, Smith, M. D., Clinical Professor of Otology, Jefferson Medical College, Philadelphia. With 244 colored figures on 39 lithographic plates, 99 text illustrations, and 292 pages of text. Philadelphia and London: W. B. Saunders & Co. 1902. [Cloth, $3.00 net.]

Saunders's Medical Hand-Atlases. Atlas and Epitomé of Operative Surgery. By Dr. Otto Zuckerkandl, Privatdocent in the University of Vienna. From the second revised and enlarged German edition. Edited,with additions, by J. Chalmers DaCosta, M. D., Professor of the principals of Surgery and of Clinical Surgery, Jefferson Medical College, Philadelphia, etc. Second edition, thoroughly revised and greatly enlarged. With 40 colored plates, 278 text illustrations and 410 pages of text. Philadelphia and London: W. B. Saunders & Co. 1902. [Cloth, $3.50 net.]

The Mental State of Hystericals. A Study of Mental Stigmata and Mental Accidents. By Pierre Janet, Litt. D., M. D., Professor of Philosophy at the College Rollin. With a preface by Professor J. M. Charcot. Translated by Caroline Rollin Corson. Octavo, pp. 553. New York and London: G. P. Putnam's Sons. 1902.

Manual of Childbed Nursing, with Notes on Infant Feeding. By Charles Jewett, A. M., M. D., Sc. D., Professor of Obstetrics and Diseases of Women in the Long Island College Hospital. 12mo, pp. 84. Fifth edition, revised and enlarged. New York: E. B. Treat & Co. 1902. [Price, 80 cents.]

Genito-Urinary Diseases and Syphilis. For students and practitioners. By Henry H. Morton, M. D., Clinical Professor of Genito-urinary Diseases in the Long Island College Hospital; Genito-urinary Surgeon to the Long Island College and Kings County Hospitals and the Polhemus Memorial Clinic. Illustrated with half-tones and full-page color plates. Pages xii—372. Philadelphia: F. A. Davis Co., Publishers. 1902. [Price, extra cloth, $3.00 net, delivered.]

The Diagnosis of Surgical Diseases. By Dr. E. Albert, late Director and Professor of the first Surgical Clinic at the University of Vienna. Authorised translation from the eighth enlarged and revised edition, by Robert T. Frank, A. M., M. D. Octavo, pp. 427. With 53 illustrations. New York: D. Appleton & Co. 1902. [Price, cloth, $5.00.]

The Diagnosis of Nervous and Mental Diseases. By Howell T. Pershing, M. Sc., M. D., Professor of Nervous and Mental Diseases in the University of Denver; Neurologist to St. Luke's Hospital, etc. Small octavo, Illustrated. Philadelphia: P. Blakiston's Son & Co. 1901. [Price, $1.25.]

A Reference Handbook of the Medical Sciences. Embracing the Entire Range of Scientific and Practical Medicine and Allied Science. By various writers. A new edition, completely revised and rewritten. Edited by Albert H. Buck, M. D., New York City. Volume IV. E. R. G.—I. N. F. Illustrated by chromolithographs and 859 half-tone and wood engravings. Nine volumes, imperial 8vo. New York: William Wood & Co. 1902. [Price, muslin, $6.00 per volume; leather, $7.00 per volume; half-morocco, $8.00 per volume.]

The Practical Medical Series of Year Books. Ten volumes. Issued monthly under the general editorial charge of Gustavus, P. Head, M. D., Professor of Laryngology and Rhinology in the Chicago Post-Graduate Medical School. Volume IV. Gynecology. Edited by Emilius C. Dudley, A. M., M. D., Professor of Gynecology, Northwestern University Medical School; Gynecologist to St. Luke's and Wesley Hospitals, Chicago. With the Collaboration of William Healy, A. B., M. D. 12mo, pp. 212. Illustrated. Chicago: The Year Book Publishers. 1902. [Price, $1.25.]

A Handbook of Appendicitis. By A. J. Ochsner, M. D., Professor of Clinical Surgery, College of Physicians and Surgeons, Medical Department of the University of Illinois; Surgeon to the Augustana Hospital, etc. Pp., 182. Illustrated. Chicago: G. P. Engelhard & Co. 1902. [Price, $1.00 net.]

